# Lahore Veterinary College

**Published:** 1891-07

**Authors:** 


### VETERINARY COLLEGE NOTES.

*The Lahore Veterinary College.*—Sir James Lyall distributed the prizes and diplomas at the Lahore Veterinary College on Wednesday May 6th; assisting by their presence were many officers of the army, and magistrates of Lahore.

The Principal (Mr. Nunn) read the following address :—

In the first place I have to thank you in the name of the students and teachers of the College for your presence, and for myself it is extremely gratifying in this the first year that I have been Principal of the Lahore

Veterinary College, to see that the interest taken in the institution has not fallen off, as is proved by your attendance here to-day

I must apologize for the makeshift accommodation I have to place at your disposal, as the building you now are in is only the side used for examining horses, but I trust that such of you as next year will honor us with your presence; will be received in a more suitable manner in the new lecture theatre which will, I trust, be finished before the coming winter session. Nevertheless, when I contrast the appearance of the College to-day with what it was when I first became connected with it in 1882—when it was only a collection of a few grass huts—I cannot help admiring the energy and perseverance of my predecessor, George Kettlewell, Esq., who now holds the appointment of Inspecting Veterinary Surgeon to the Madras Army, and to whom is due the credit of the present popularity and efficiency of this institution. That the Veterinary College is popular amongst the inhabitants of Lahore can be seen by the hospital books, which show that 352 horses have been treated this year against 336 last, an increase of 16, and in the out-patient department 471 against 387 last year, or an increase of 84.

The cattle practice has developed largely, 517 patients against 431 last year, or an increase of 85. Both this and the number of in-door equine patients would have been much larger, only for nearly three months at the busiest time of the year, the cattle hospital, consisting of twelve boxes, which is also used for free equine patients, was in the hands of the builders undergoing repairs.

This cattle practice is a most important part of the training of students whose future career will be in district work, and in connection with it a new book on an important branch of bovine pathology has been prepared in Urdu, by Sirdar Shah, teacher of the subject, and which has been made one of the text books.

I trust that before long a new sphere of usefulness will be opened out. In the new buildings which are being erected is included a laboratory, and when this is ready and fitted with the necessary apparatus, I hope to be able to commence a series of experiments into the nature of some of the more obscure diseases of animals in India, especially as to the cultivation of vaccine for protective inoculation after the method of Pasteur. Should these be carried out, I hope that from Lahore we may be able to contribute some little towards the question that is attracting increased attention daily in the medical and scientific world, *viz.*, the relationship of infectious and contagious diseases between animals and man. That this is a vast and almost untrodden field of research is shown by the experiments of Signor Thomasseni in Italy, which seem to point out that, if not derived from—at all events there is some close connection between tetanus in the horse and man. The experiments of Dr. Klein in London, as to the possibility of scarlatina in the human being, being derived from the cow, and the observations of Dr. Davison in Buenos Ayres, in South America, as to diptheria in man and fowls, all point to the possibility of brilliant results being obtained in this direction. In our own profession I trust to be able to make some inquiries into the outbreaks of the disease known as anthrax that is at times so prevalent and causes such loss amongst Cavalry horses in certain stations in India. I myself have, perhaps, peculiar views on this subject, but without troubling you with my reasons, which would be too complicated to explain here, all I will say is that I have great doubts as to this disease being anthrax at all. Some few years ago I was employed in investigating a fatal disease amongst horses in Natal and the South African Colonies, known as Harn Gickners, and which was always looked on as being anthrax. The experiments conducted in a proper manner and with suitable apparatus proved that this was not the case. And that by adopting certain precautions the number of seizures was lessened, and that by following out a certain line of treatment, a larger percentage of animals recovered. Furthermore it was shown that the elaborate system of disinfection and isolation was useless, and a considerable saving in money effected in that way only; and, as I have before said, I have reasons to believe that, were a proper series of experiments instituted, which I hope to be able to do, that the same would be found to be the case in India.

With regard to the passed pupils of the College, all I can say is that, as their usefulness gets known to the public, there is an increasing demand for their services. Just before this last examination I had on my list applications for Veterinary Assistants from differents parts of India, Central Provinces and Burma, and there was not a single unemployed man I could send to fill them. Furthermore two of the last passed men have been engaged and are now on their way to Manipur.

I can speak from practical experience of their usefulness in the field, as during the recent China Lushai expedition, I had a number of Veterinary Assistants under my orders. Most of them had been educated at the Lahore College, and I may safely say that without them it would have been absolutely impossible to have worked the transport of the expedition. Out of the present year's students 44 presented themselves for examination, 20 obtained certificates as first, and 17 as second class Veterinary Assistants, 7 failing to satisfy the examiners. I must again thank Your Honor and gentlemen for favoring us with your presence at the distribution of diplomas and prizes, which I am certain will render them all the more valuable in the eyes of the recipients.

His Honor then presented the prizes to the successful candidates. Addressing the students, he pointed out that there was no lack of employment for Veterinary Assistants. The inhabitants of Lahore appreciated the advantages of the College but a wider view should be taken of it, and it should be looked on as a means of spreading knowledge over the province and Northern India. He also testified to the merits and abilities of Mr. Kettlewell. And with regard to the new buildings in the College and the proposed laboratory, said he hoped that it would be established and the experiments that the Principal mentioned that he proposed to institute, would be successful, and that useful and scientific results of interest would come of them, especially with regard to the relationship of disease between human beings and animals.

His Honor's speech was translated and given in vernacular by Dr. Rabim Khan, Khan Bahadur, Lecturer on Materia Medica at the Veterinary College.

The teachers were presented to His Honor by the Principal, and after inspection of the new lecture theatre and laboratory the proceedings terminated.

